# ﻿A new synonym of *Mussaenda* (Rubiaceae) from Vietnam

**DOI:** 10.3897/phytokeys.266.165871

**Published:** 2025-11-27

**Authors:** Wei-Yuan Huang, Tie-Yao Tu, Khang Sinh Nguyen, Van Son Dang, Ting-Ting Duan

**Affiliations:** 1 College of Coastal Agricultural Sciences, Guang Dong Ocean University, Zhanjiang CN-524088, Guangdong, China Guang Dong Ocean University Zhanjiang China; 2 South China Botanical Garden, Chinese Academy of Sciences, Guangzhou CN-510650, Guangdong, China South China Botanical Garden, Chinese Academy of Sciences Guangzhou China; 3 Institute of Ecology and Biological Resources, Vietnam Academy of Science and Technology, 18 Hoang Quoc Viet, Cau Giay, Ha Noi, Vietnam Institute of Ecology and Biological Resources, Vietnam Academy of Science and Technology Ha Noi Vietnam; 4 The VNM Herbarium, Institute of Tropical Biology, Vietnam Academy of Science and Technology, 85 Tran Quoc Toan Street, District 3, Ho Chi Minh City, Vietnam Vietnam Academy of Science and Technology Ho Chi Minh City Vietnam

**Keywords:** *Mussaenda* Burm. ex L., *Mussaenda recurvata* Naiki, Tagane & Yahara, new synonym, taxonomy

## Abstract

Based on examination of type specimens and original literature, it has been determined that *Mussaenda recurvata* Naiki, Tagane & Yahara and *Mussaenda reflexisepala* Tao Chen & Duy are conspecific. Therefore, *M. reflexisepala* is hereby treated as a synonym of *M. recurvata*. A new Chinese name is proposed for *M. recurvata*: Juǎn è Yù yè Jīn huā.

## ﻿Introduction

*Mussaenda* Burm. ex L. (Rubiaceae, Dialypetalanthoideae, Mussaendeae) comprises approximately 135 species, predominantly distributed in Asia and Africa, with Asia representing the centre of diversity (Alejandro et al. 2005; Chen and Taylor 2010; Razafimandimbison and Rydin 2024). Species in this genus are climbing or scandent shrubs or lianas, with Vietnam hosting around 30 species (Pitard 1923). The current classification primarily relies on characters, such as leaf morphology (shape, venation, pubescence), stipule morphology, calycophyll morphology, the relative lengths of calyx tubes and lobes and the shapes of calyx and corolla lobes (Deng 2007; Zhang et al. 2024).

**Figure 1.**
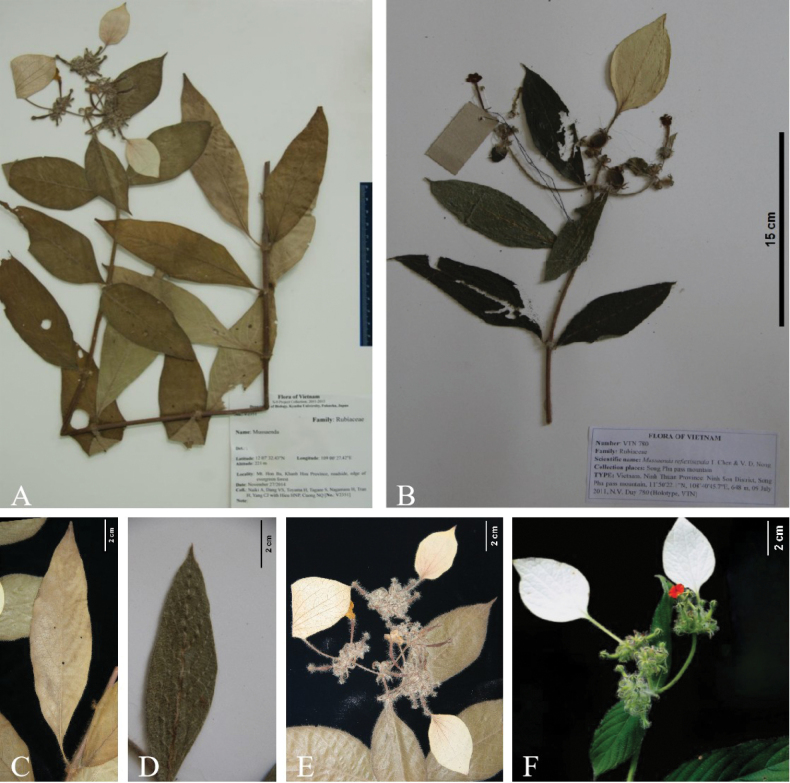
Morphological comparison of *Mussaenda recurvata* and *M. reflexisepala*. **A.** Isotype of *M. recurvata* (Toyama et al. V2351, VNM); **B.** Holotype of *M. reflexisepala* Tao Chen & Duy (N.V. Duy 780, VTN); **C.** Leaf of *M. recurvata*; **D.** Leaf of *M. reflexisepala*; **E.** Flowers of *M. recurvata*; **F.** Flowers of *M. reflexisepala*. **A, C, E.**, Toyama et al. V2351 (VNM); **B, D, F.**, N.V. Duy 780 (VTN).

In 2017, Naiki et al. described *Mussaenda recurvata* Naiki, Tagane & Yahara (Fig. 1A) from Khanh Hoa Province, Vietnam, with the type locality at Hon Ba Nature Reserve (Naiki et al. 2017). It was distinguished from *M. longipetala* H.L.Li by its strongly recurved calyx lobes, shorter corolla tube and ovate corolla lobes, contrasting with the straight calyx lobes, longer corolla tube and lanceolate corolla lobes of the latter species.

Later that year, N. V. Duy and T. Chen described *Mussaenda reflexisepala* Tao Chen & Duy (Fig. 1B) from Ninh Thuan and Khanh Hoa Provinces, Vietnam. The type locality is in Ninh Son District, with the distribution extending to Hon Ba Nature Reserve (Nong and Chen 2017). The protologue distinguished it from *M. caudatiloba* D.Fang, based on its strongly recurved calyx lobes, yellow to orange or reddish-orange corolla and ovate to broadly obovate corolla lobes, versus straight calyx lobes, yellow corolla and triangular-lanceolate corolla lobes in *M. caudatiloba*.

**Figure 2.**
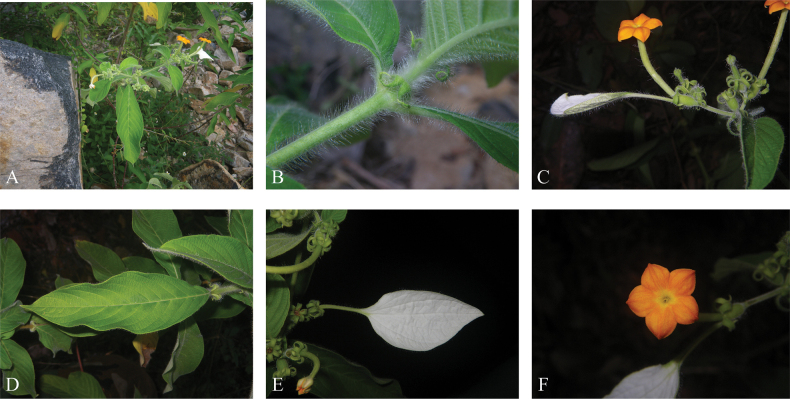
*Mussaenda recurvata* in the field. **A.** Flowering plant in habitat; **B.** Stipules; **C.** Inflorescence; **D.** Leaves; **E.** Calycophyll; **F.** Flower.

Comparative analysis of type specimens and protologues conducted during our taxonomic study of *Mussaenda* in Indochina revealed no diagnostic differences in leaf, flower, fruit morphology or indumentum between *M. recurvata* and *M. reflexisepala* (Fig. 1; Table 1). Both exhibit densely pubescent twigs and leaves, strongly recurved calyx lobes with no consistent variation in leaf shape (Fig. 1C, D), calycophyll morphology or corolla lobe shape (Fig. 1E, F). Notable protologue differences, such as elliptic-lanceolate leaves in *M. recurvata* versus narrowly elliptic ones in *M. reflexisepala* (1.2–13.3 cm × 0.8–4.5 cm) and corolla lobe colour (orange-yellow vs. yellow to reddish-orange) were assessed through field surveys (Fig. 2) and direct examination of the isotype of *M. recurvata* (Fig. 1A) and the holotype of *M. reflexisepala* (Fig. 1B). Our observations confirmed that *M. reflexisepala* also bears elliptic-lanceolate leaves (Fig. 1D) with mature dimensions consistent with *M. recurvata*. The broader leaf-size range of *M. reflexisepala* likely reflects inclusion of immature leaves. Under natural light, both taxa exhibit orange corolla lobes with consistently cuspidate apices, contradicting the colour variation suggested in earlier reports. Other characters, such as indumentum density, stipule length, number of secondary veins, calycophyll size and calyx lobe morphology show negligible variation. All observed features of *M. reflexisepala* fall within the morphological range of *M. recurvata*, supporting their taxonomic unification (Table 1).

**Table 1.** Morphological comparison between *M. recurvata* and *M. reflexisepala*.

Trait	*M. recurvata*	*M. reflexisepala*
**Twig pubescence**	Densely white villose	Densely white villose
**Leaf shape**	Elliptic or elliptic-lanceolate	Elliptic
**Leaf pubescence**	Pilose and puberulous	White pilose
**Leaf size**	8–14 cm × 2.5–5 cm	1.2–13.3 cm × 0.8–4.5 cm
**Secondary veins**	9–13 pairs	8–10 pairs
**Stipule**	Triangular-lanceolate, deeply bifid, 5–8 mm long	Triangular-lanceolate, deeply bifid, 7–10 mm long
**Calyx lobes**	5, narrowly triangular-lanceolate,	5, narrowly lanceolate, 5–7 mm long,
	4.5–15 mm long, apex acute, strongly recurved, covered with puberulous hairs on both surfaces	apex acute, strongly recurved, covered with pilose hairs on both surfaces
**Calycophylls**	Ovate or elliptic, both sparsely pilose and densely puberulous on both surfaces,	Ovate or irregularly ovate, sparsely pilose adaxially, villose abaxially,
	17–65 mm × 7–54 mm	30–60 mm × 25–45 mm
	apex acute to acuminate, base acute to cuneate	apex acuminate, base broadly cuneate
**Corolla tube**	18–26 mm long	20–25 mm long
	Covered with both pilose and puberulous hairs outside, inside densely covered with yellow pilose hairs	Densely covered with white villose hairs outside, inside densely covered with yellow clavate pairs
**Corolla lobes**	Orange-yellow to orange	Yellow to orange or reddish-orange
	ovate	ovate to broadly obovate
	5–7 mm × 4–5.5 mm	5–6.5 mm long
**Fruits**	15–19 mm × 10–11 mm	12–20 mm × 8–11 mm

Both taxa are endemic to Vietnam and share overlapping distributions. *M. recurvata* has only been recorded from Khanh Hoa Province, while all paratype specimens of *M. reflexisepala* were collected from Hon Ba Nature Reserve and adjacent areas. Despite overlapping morphological and geographical features, the protologue of *M. reflexisepala* did not reference *M. recurvata*, possibly due to the 45-day publication interval between their respective descriptions (17 November 2017 vs. 31 December 2017), which may have prevented effective nomenclatural comparison.

Based on these findings, we conclude that the two names represent a single species. We therefore reduce *M. reflexisepala* to synonymy under *M. recurvata*, following the International Code of Nomenclature (Turland et al. 2018). We propose the Chinese vernacular name “Juǎn è Yù yè Jīn huā” (卷萼玉叶金花) for *M. recurvata*.

## ﻿Taxonomic treatment

### ﻿ Mussaenda recurvata

Taxon classificationPlantaeGentianalesRubiaceae

Naiki, Tagane & Yahara, Phytotaxa 328: 167. 2017.

1E3AF5ED-247A-5C1A-B118-4A701A28D1DF

Fig. 2

≡ Mussaenda reflexisepala T. Chen & V. D. Nong, syn. nov., “Harvard Papers in Botany” 22: 127. 2017. Type. Vietnam. Ninh Thuan Province: Ninh Son District, Song Pha Pass Mountain, alt. 648 m, 11°50'22.1"N, 108°40'45.7"E, 09 July 2011, N.V. Duy 780 (holotype: **VTN**!; isotype: **SZG**!).

#### Type.

Vietnam. Khanh Hoa Province: • Hon Ba Nature Reserve, alt. 221 m, 12°07'32.43"N, 109°00'27.42"E, 27 November 2014, A. Naiki, V. S. Dang, H. Toyama, S. Tagane, H. Nagamasu, H. Tran & C. J. Yang V2351 (holotype: **KYO**!; isotype: **VNM**!).

#### Description.

Shrubs, evergreen, scandent, 1.5–5 m tall, twigs terete, covered with both pilose (1.5–4.5 mm long) and densely puberulent (0.1–0.3 mm long) hairs on younger branches, most of the pilose hairs caducous, but the puberulent hairs remaining on older branches. ***Stipules*** triangular or triangular-lanceolate, 5–8 × 3–5 mm [including lobes], recurved outwards, deeply bilobed at apex, lobes narrowly triangular to linear, 3–4 × 1–1.5 mm, covered with densely pilose and puberulous hairs on both surfaces. ***Petioles*** 2–10 mm long, densely white villose. ***Leaves*** opposite, chartaceous, blade elliptic or elliptic-lanceolate, 8–14 cm × 2.5–5 cm, base cuneate or attenuate into petiole, covered with both white pilose and puberulous (0.3–0.5 mm long) hairs on both surfaces, especially denser on the mid-rib and secondary veins, apex acute or acuminate, mid-rib and secondary veins flat adaxially, prominent abaxially. ***Secondary veins*** 8–12 pairs, ascending at an angle of 50–70° from mid-rib, curved to the margin. ***Inflorescences*** terminal and sometimes also in axils of uppermost leaves in a branch, cymose, usually regularly dichotomous, sometimes secondary axis poorly elongating, peduncles 1–3.5 cm long, secondary axis up to 1.5 cm long, ***Bracts*** lanceolate or narrowly triangular, 1.5–3.5 mm long, densely white villose, sometimes slightly bilobed. ***Flowers*** sessile or pedicellate, pedicel up to 2 mm long, covered with both pilose and puberulous hairs. ***Calyx*** tube urceolate, 2.5–4 × 2–3 mm in short-styled flowers, 3–6 × 2.5–3.5 mm in long-styled flowers, densely covered with both pilose and puberulous hairs outside, lobes 5, narrowly triangular, 4.5–15 × 1–2 mm, apex acute, covered with puberulous hairs on both sides and pilose at the margin, strongly recurved. ***Calycophyll*** white on upper surface, pale green on lower surface, blade ovate, irregularly ovate or elliptic, both sparsely pilose and densely puberulous on both surfaces, chartaceous, 1.7–6.5 × 0.7–5.4 cm, base acute to cuneate, apex acute to acuminate, stipe 8–35 mm long. ***Corolla*** salverform, 9–15 mm in diam., tube pale green, 18–26 mm long in short-styled flowers, 18.5–22.5 mm long in long-styled flowers, 1–1.5 mm in diam. at base, ca. 2.5 mm in diam. at the widest part [anther-inserted part] in both long-styled and short-styled flowers, outside covered with both pilose and puberulous hairs, inside upper 2/3 to 3/4 part densely covered with upward yellow pilose hairs, sparsely so on the remaining portion. ***Lobes*** 5, reduplicate-valvate in bud, orange-yellow to orange on upper surface, pale orange on lower surface, ovate, 5–7 × 4–5.5 mm, basal 1/4 to 2/5 portion fused with adjacent lobes, cuspidate at apex, cusp 0.5–0.7 mm long. ***Stamens*** 5, inserted in middle to upper part of the corolla tube, included, filaments adnate to the tube, 9.5–15.5 mm long in short-styled flowers, 7.5–11 mm long in long-styled flowers, anthers dorsifixed, narrowly oblong, 4.5–6.5 mm long in short-styled flowers, narrowly lanceolate 2.5–4.5 mm long in long-styled flowers, pollen absent in the anthers of long-styled flowers, ovary 2-celled, ovules numerous in each cell, style 5–10 mm long in short-styled flowers, 10–14 mm long in long-styled flowers, stigma 2-lobed, lobes linear, 6–8.5 mm long, included in short-styled flowers, 8–12.5 mm long, slightly exserted in long-styled flowers. ***Fruits*** green when young, baccate, ellipsoid, 15–19 mm long, 10–11 mm in diam., covered with both pilose and puberulous hairs, with strongly recurved calyx lobes persistent. ***Seeds*** dark brown, numerous, lenticular, ca. 0.5 × 0.3 mm, ca. 0.1 mm thick, testa foveolate.

#### Distribution and habitat.

Endemic to Vietnam, distributed in Khanh Hoa, Ninh Thuan and Lam Dong Provinces. It grows sporadically on scrub roadside of the evergreen secondary forest. In Hon Ba Nature Reserve, this species is occasionally encountered, but easily observed along roadsides at elevations of 200–900 m.

#### Specimens examined.

**Vietnam.** • **Khanh Hoa**, Nha Trang, 22 January 1923, Poilane 5399 (P barcode 06590811, 06590813); • Nha Trang, 1927, J. & M.S. Clemens 4466 (P barcode 04010128, U barcode 0041557); • Nha Trang, 19 September 2013, T. Y. Tu 1099 (ISBC); • Hon Ba Nature Reserve, 30 November 2004, D. Soejarto et al. 13622 (P barcode 01152527); • 12°07'52.32"N, 109°01'31.8"E, alt. 120 m, Hon Ba Nature Reserve, 19 February 2014, H. Toyama et al. V799 (VNM); • 12°06'40.06"N, 108°58'58.75"E, alt. 622 m, Hon Ba Nature Reserve, 21 November 2014, H. Toyama et al. V1946 (VNM); • 12°13'29.23"N, 108°46'46.42"E, alt. 877 m, **Lam Dong**, Da Lat, 9 February 1914, A. Chevalier 80614 (P barcode 04540942, 04010166).

## Supplementary Material

XML Treatment for Mussaenda recurvata

## ﻿Additional information

### Use of AI

No use of AI was reported.

### Author ORCIDs

Wei-Yuan Huang https://orcid.org/0009-0009-5895-6447

Tie-Yao Tu https://orcid.org/0000-0001-7385-008X

Khang Sinh Nguyen https://orcid.org/0000-0001-5171-4140

Van Son Dang https://orcid.org/0000-0001-8681-4141

Ting-Ting Duan https://orcid.org/0000-0003-2140-9967

### Data availability

All of the data that support the findings of this study are available in the main text.
